# Medical Assistance in Dying: the opinions of medical trainees in Newfoundland and Labrador. A cross- sectional study

**Published:** 2019-11-28

**Authors:** Robert McCarthy, Melanie Seal

**Affiliations:** 1 Faculty of Medicine, Memorial University, Newfoundland, Canada

## Abstract

**Background:**

Medical Assistance in Dying (MAiD) was legalized in Canada in 2016. As future physicians, medical trainees may face decisions regarding MAiD. Although many publications exist internationally, Canadian data is limited in the peer-reviewed literature, particularly following legalization. The purpose of this study is to determine the opinions of medical trainees in Newfoundland and Labrador regarding MAiD, and the factors that impact these views.

**Methods:**

A survey was distributed to all under- and postgraduate medical trainees at Memorial University (N=570), the only medical school in Newfoundland and Labrador. The survey collected demographic information and opinions regarding MAiD. Respondents were divided into groups based on demographic characteristics, and their responses analyzed using non-parametric statistics.

**Results:**

The survey was completed by 124 trainees. Ninety percent of respondents agreed with the legalization of MAiD in Canada and nearly 60% stated they would perform the procedure for their patients. Several factors influenced the opinions of medical trainees, including level of training and religious affiliation. Trainees also favored detachment from the MAiD process.

**Interpretation:**

Medical trainees in Newfoundland and Labrador are largely in favor of MAiD. This may highlight the importance of emphasizing MAiD within medical curricula, so that trainees are adequately informed and prepared for this new aspect of medical care upon joining independent practice.

## Abstract (fr)

**Contexte:**

L’aide médicale à mourir a été légalisée au Canada en 2016. En tant que futurs médecins, les apprenants en médecine devront peut-être prendre des décisions concernant l’aide médicale à mourir. Bien qu’il existe plusieurs publications au niveau international, les données canadiennes sous forme d’articles évalués par des pairs sont limitées. Cette étude vise à connaître les opinions des apprenants en médecine de Terre-Neuve-et-Labrador concernant l’aide médicale à mourir, et déterminer les facteurs qui influencent leurs points de vue.

**Méthodes:**

Un sondage a été distribué à tous les étudiants en médecine et résidents de l’Université Memorial (N=570), la seule faculté de médecine à Terre-Neuve-et-Labrador. Ce sondage a recueilli des informations démographiques et des opinions concernant l’aide médicale à mourir. Les personnes interrogées ont été divisées en groupes établis selon des caractéristiques démographiques, et leurs réponses ont été analysées à l’aide de statistiques non paramétriques.

**Résultats:**

124 apprenants ont complété le sondage. 90 pour cent des personnes interrogées étaient en faveur de la légalisation de l’aide médicale à mourir au Canada et près de 60 % ont indiqué leur intention d’effectuer cette intervention auprès de leurs patients. Plusieurs facteurs ont influencé les opinions des stagiaires en médecine, notamment le niveau de formation et l’affiliation religieuse. Les stagiaires ont également favorisé le détachement face au processus d’aide médicale à mourir.

**Interprétation:**

Les apprenants en médecine de Terre-Neuve-et-Labrador sont très favorables à l’aide médicale à mourir. Ces résultats font ressortir l’importance d’intégrer l’aide médicale à mourir aux programmes d’études en médecine pour bien informer et préparer les stagiaires à cette nouvelle dimension des soins médicaux avant de débuter leur pratique.

## Introduction

Physician-assisted death has been a common topic in the medical community for decades.^1^ Internationally, legislation exists in a few countries allowing patients who meet specific inclusion criteria to end their lives through medical means. In Canada, the prohibition on physician-assisted dying was unanimously struck down in the Supreme Court of Canada (SCC) on February 6, 2015.^2^ As of June 17, 2016, amendments to the Criminal Code of Canada and other related Acts were completed and Medical Assistance in Dying (MAiD) was legalized in this country.^3^

Several studies have reported the opinions of medical trainees towards MAiD, some including those of Canadian trainees.^4,5^ However, the data for these Canadian studies were collected before the official legalization of MAiD. Despite this, Bator et al demonstrated strong support for MAiD among Canadian medical students, who felt that patient autonomy was the key ethical principle driving their view.^4^ The authors also found that religious ties reduced support for MAiD, and that students were less likely to support MAiD for patients with mental illnesses.^4^ This cohort expressed a need for additional training in MAiD, specifically around medical-legal issues, communication skills and technical aspects of the MAiD process.^4^ Spicer et al assessed the opinions of residents regarding MAiD and concluded that while most residents would be willing to provide MAiD for eligible patients, they felt that more formal training around MAiD and palliative care was required prior to doing so.^5^

From an international perspective, several studies suggest that religiosity^6-11^ and female gender^6-8^ are associated with reduced support for MAiD. Progression through medical training also influences perspectives, with senior students more apprehensive about providing lethal prescriptions than their juniors.^12^ Similarly, attending physicians are less accepting than trainees.^1,7,13^^-^^15^ Other factors that may affect the opinions of trainees include legality^9^ and exposure to palliative care training.^6,13^

The extent to which trainees wish to be involved in MAiD is also described in the literature. This refers to their willingness to administer a lethal drug, provide a prescription for self-administration or refer to another healthcare provider for MAiD. Overall, trainees often favor self-administration by the patient over injecting the medication themselves.^4,15^^-^^17^ In the medical community, assisted death may be viewed by some as contradictory to the “professional ethos”^15^ of a physician, misaligned with the typical goals of sustaining life.^15^^-^^16^ Other reasons favoring detachment from the process include the potential for creating a “slippery slope”,^15^ along with medical- legal concerns among physicians.^17^

In Newfoundland and Labrador, the site of our study, the current framework for MAiD deems the process acceptable for patients 18 years of age and older who (1) have an irremediable medical condition that (2) causes enduring and intolerable suffering and (3) whose death is reasonably foreseeable.^18^ The patient must demonstrate capacity and decide to proceed without coercion. The process requires involvement of two eligible healthcare providers (physicians or nurse practitioners) who each must independently determine that the patient is an appropriate candidate for MAiD. While trainees may be involved in the process for learning purposes, they are not permitted to act as an independent assessor.

Medical trainees may face decisions regarding MAiD throughout their careers and their opinions towards this practice are relevant when shaping the regulatory framework that will govern it. Since MAiD was legalized in Canada, the opinions of medical trainees are largely absent in the peer-reviewed literature. Therefore, the purpose of our study is to gauge the opinions of medical trainees regarding MAiD in Newfoundland and Labrador, and to propose some potential demographic factors that may influence these views. Since the Canadian literature suggests that a gap exists regarding MAiD training,^4,5^ we will also explore connections between our findings and the potential role for additional education.

## Methods

We developed a survey tool (Appendix A) collecting demographic information, as well as responses to several statements pertaining to MAiD using a five- point Likert scale ranging from *strongly disagree* to *strongly agree*. Our objective was to explore the opinions of trainees at Memorial University based on the relevant findings in the literature.

Following approval from the Newfoundland and Labrador Health Research Ethics Board, we distributed surveys via university e-mail to all students and residents enrolled in the undergraduate MD and postgraduate degree programs. Participants received e-mail reminders approximately one week following survey distribution. Participation in the study was voluntary and completion of the survey represented implied consent. All responses were anonymous.

To obtain an overall impression of the attitudes of participants regarding MAiD, we used frequency counts to analyze the dataset. Although we collected specific information regarding many demographic factors, we often combined participants into broader categories to preserve their anonymity. We performed non-parametric statistical analysis (Mann-Whitney U test and Kruskal-Wallis ANOVA) using SPSS software (version 24.0) to assess between-group differences. A p-value of less than 0.05 denoted statistical significance.

## Results

We distributed the survey to approximately 570 medical trainees at Memorial University. 124 trainees completed it, yielding an overall response rate of 22%. The specific response rates were 24.7% among students and 18% among residents. Table 1 outlines respondent demographics. Overall, the population of respondents was comprised of 63.7% students and 36.3% residents, with most medical students in the first two years of the four-year undergraduate program. Among the resident cohort, approximately half were in the first two years of their training.

**Table 1:** Demographics of study participants (N=124)

Level of Training	Students	63.7%
	Residents	36.3%
Gender	Male	33.1%
	Female	66.9%
Age	20-29	75.8%
	30+	24.2%
Religion	Identified Religious Affiliation	45.5%
	No Religious Affiliation	54.5%
Province of Origin	Atlantic Canada	75.0%
	Other Canada	21.8%
	International	3.2%
Population of Hometown*	Small (1-29,999)	43.5%
	Medium (39,999 –99,999)	10.5%
	Large (100,000+)	46.0%
Undergraduate Degree	Bachelor of Science	76.4%
	Healthcare	9.8%
	Other	13.8%

**Fig 1:**
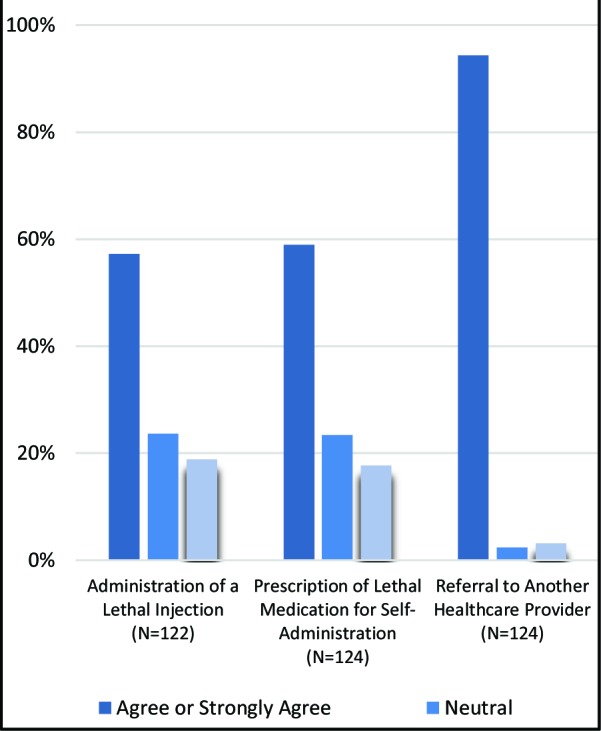
Favorability of study participants towards extent of involvement in MAiD

Participants largely supported MAiD legalization, with 89.5% of respondents selecting “agree” or “strongly agree”. Trainees more frequently favored referral for MAiD rather than direct involvement:, 57.3% agreeing to administer a lethal drug, 58.9% agreeing to prescribe a drug for self-administration and 94.4% supporting referral to another provider. (Fig. 1)

When asked about the likelihood that they would personally choose MAiD if they were diagnosed with a terminal illness, less than half of respondents agreed (47.6%), with an additional 43.5% selecting “neutral”. Interestingly, even among the subgroup of trainees that favored legalization, only 51.3% agreed that they would personally pursue MAiD if they were terminally ill.

**Fig. 2:**
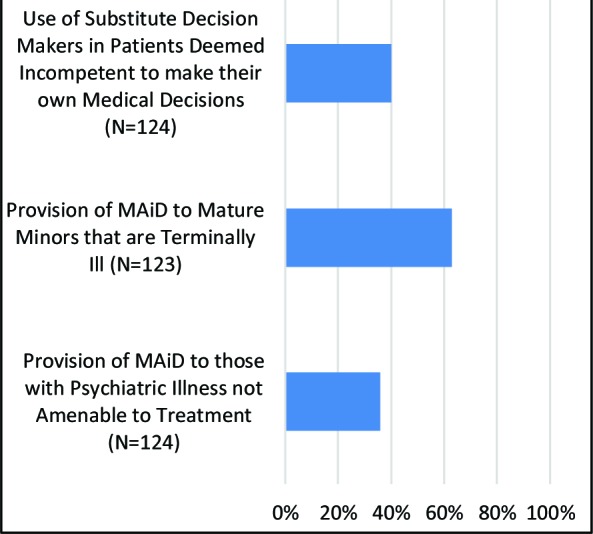
Percentage of respondents selecting “agree” or “strongly agree”

Our survey also aimed to address some of the more contentious issues surrounding MAiD, including the use of Substitute Decision Makers (SDMs) and provision to the mentally ill and mature minors. The percentage of participants expressing support for MAiD in these circumstances is illustrated in Figure 2. Much of the cohort (63.4%) supported the provision of MAiD to mature minors, however, fewer than half of them supported MAiD for the mentally ill or the use of SDMs.

**Fig. 3:**
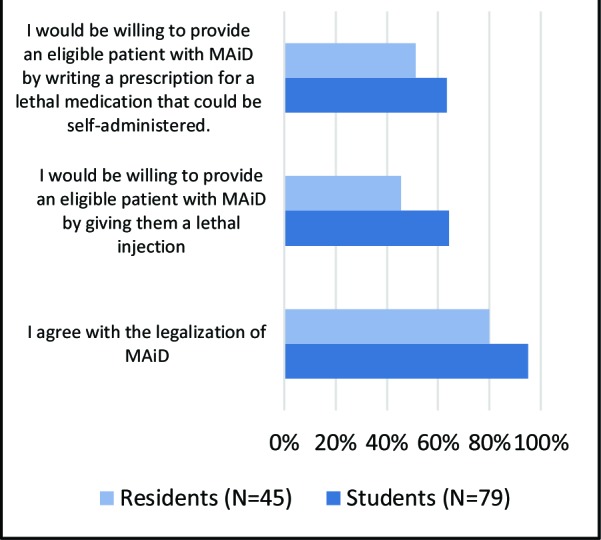
Differences in opinion between students and residents regarding MAiD

We also divided participants into groups based on demographic parameters, yielding several noteworthy between-group differences. When categorized based on level of training, students versus residents, the difference in mean-rank values of several survey questions were statistically significant (p<0.05). Students (94.9%) were more likely to agree with MAiD legalization than residents (80.0%). Furthermore, students were also more likely to agree with both administration of medication and the writing of lethal prescriptions. (Fig. 3). No significant differences in mean-rank values were observed among other survey questions.

Participants were also grouped based on religious affiliation, with 56 participants declaring religious ties and 67 having no religious affiliation. Higher mean rank scores were observed in the religiously unaffiliated group across all questions regarding MAiD. For instance, 97.0% of participants with no religious affiliation agreed with MAiD legalization, compared to 82.4% of those affiliated with a religious faith. Additionally, participants without religious attachments were more agreeable to each form of provision explored, as well as the use of SDMs. They were also more likely to support MAiD for the mentally ill and mature minors, and to express a personal interest in MAiD if they were terminally ill.

Several other factors were considered, including age, gender, undergraduate degree and population of a participant’s city or town of origin. No statistically significant differences were observed between groups based on these parameters for any of the survey questions.

## Discussion

Overall, medical trainees at Memorial University are largely supportive of MAiD, with nearly 90% of respondents agreeing with its legalization. Regarding extent of involvement, trainees prefer an indirect role in the process, with most agreeing to refer patients to another healthcare provider for MAiD. More direct approaches, such as the administration of a lethal medication or prescribing one for self-administration were less favorable, which is consistent with other published literature.^15^^-^^17^ This is perhaps not surprising, as the concept of MAiD elicits a conflict between two fundamental ethical principles in medicine – autonomy and non-maleficence. Considering life as valuable is fundamental to the culture of medicine,^15^ and directly participating in a patient’s death can be considered a violation of moral beliefs.^17^ Interestingly, 5.6% of our population did not agree with referral to another provider, which may call their knowledge of the current regulatory framework into question, as conscientious objectors currently must refer eligible patients to another provider. This may highlight a need for further education around the local regulatory framework governing MAiD. Alternatively, these participants may disagree with the current framework, and thus be willing to refuse referring their patients regardless of the consequences.

Although trainees agree with legalization of MAiD, there is hesitancy to support contentious topics, such as the provision of MAiD to patients with psychiatric illnesses. Trainees elsewhere are also reluctant to support MAiD for patients experiencing psychological suffering.^4,7^ Moreover, staff physicians have also been apprehensive towards MAiD for patients experiencing mental illness.^19^ We postulate that this may reflect concerns about a patient’s capacity to consent, given that a comorbid mental illness can pose challenges regarding capacity assessment. A further consideration among our population is that our survey simply states, ‘psychiatric illness that is refractory to treatment’. This leaves room for interpretation from the respondent, who may be more willing to accept or deny MAiD depending on the disease and the degree of suffering that it is causing. Finally, this may reflect a frequently discussed stigma around mental illness that exists in society today. When considering the use of SDMs, our study population was also unsupportive. Concerns have been expressed previously about the potential for creating a “slippery slope” with MAiD legalization.^15^ A similar concept may be at play here, as designating SDMs introduces the potential for patient coercion and compromising patient autonomy. The ethical principle of autonomy was a key factor for Canadian medical students to support MAiD,^4^ which may corroborate this hypothesis. Finally, study participants were largely in favor of MAiD provision for mature minors, which again may highlight trainees’ respect for patient autonomy. We anticipate that mature minors would be supported with caution, perhaps in the setting of terminal illness where the patient was deemed competent to make independent medical decisions. It has been suggested that legality may also influence one’s views of MAiD,^9^ however, our results are not consistent with this. Among the three contentious issues explored, the provision of MAiD for patients with psychiatric illness received the least support, yet is the only issue that is not explicitly prohibited within the current regulatory framework.^18^

Among the study population, one’s level of training impacted their opinion of MAiD, with medical students more frequently supporting direct approaches. Similar views were expressed by medical students at another Canadian university.^4^ This illustrates that agreement with MAiD may decrease as we ascend the medical hierarchy, as demonstrated by similar studies.^1,7,12^^-^^15,20^ While these views may reflect a different moral stance among physicians and residents, some have proposed that these differences exist due to the longstanding relationships that physicians have with their patients along with generational differences that exist between groups.^15^ Our study results, however, did not demonstrate statistically significant differences in opinion based on the age of participants. Since legalization of MAiD is a recent development in Canada, senior residents are also less likely to have received formal education in the practice.^5^ As such, they may feel less comfortable offering it, which may explain the lower agreeability that we observed among this group. In another Canadian study, only 35% of residents felt as though their programs provided adequate training to make informed decisions about MAiD.^5^ This may support a need for further MAiD training within undergraduate and postgraduate medical curricula.

Having an identified religious affiliation may also impact a trainee’s attitude towards MAiD. In this study, participants with a religious affiliation were less likely to agree with all elements of MAiD addressed in the questionnaire, a finding that has been replicated elsewhere.^4,6^^-^^9^ Among the religiously affiliated, 79% were of the Christian faith. Therefore, our result may have been biased by a predominance of this belief system, as other literature has demonstrated reduced support for MAiD among Catholics.^6^ Among the subgroup of participants who identified as Christian in our study, only 22% agreed that their religion had a large impact on their everyday lives, and among this smaller subgroup, 70% still expressed support for MAiD legalization. In Newfoundland and Labrador, where the current rate of MAiD provision is below the national average, several faith-based healthcare facilities have expressed opposition toward the practice.^21^ Therefore, this difference may also be explained by the predominant views of local religious groups as opposed to those of a specific faith.

There are some limitations to our study. With a small sample size, the impact of several demographic factors, such as specific religious affiliations, type of undergraduate degree and postgraduate training program could not be addressed. Additionally, the presence of responder bias and the inclusion of participants at one medical school in one Canadian province may limit the generalizability of our results to other Canadian trainees. Finally, given that trainees currently are not permitted to be directly involved in MAiD, their responses to survey questions are hypothetical. Therefore, it is possible that, if participants were permitted to offer MAiD to patients, their opinions would change.

In conclusion, our results demonstrate that medical trainees in Newfoundland and Labrador are largely supportive of MAiD, however favour more strongly a detached role from the process. While religious affiliation and level of training may influence opinions regarding MAiD, our results also demonstrate a potential need for additional training at the undergraduate and postgraduate levels, which is consistent with other Canadian studies on the topic.^4,5^ MAiD remains a new concept in Canada and should continue to evolve both practically and theoretically. By understanding the factors that influence the opinions of future physicians, we may play a role in informing the practice of MAiD in our country.
